# Agouti-Signalling Protein Overexpression Reduces Aggressiveness in Zebrafish

**DOI:** 10.3390/biology12050712

**Published:** 2023-05-13

**Authors:** Ana Rocha, Alejandra Godino-Gimeno, Josep Rotllant, José Miguel Cerdá-Reverter

**Affiliations:** 1 Centro Interdisciplinar de Investigação Marinha e Ambiental (CIIMAR), Terminal de Cruzeiros do Porto de Leixões, 4450-208 Matosinhos, Portugal; 2 Control of Food Intake Group, Department of Fish Physiology and Biotechnology, Instituto de Acuicultura de Torre de la Sal, IATS-CSIC, 12595 Castellon, Spain; 3 Instituto de Investigaciones Marinas, Consejo Superior de Investigaciones Científicas (IIM-CSIC), 36208 Vigo, Spain

**Keywords:** ASIP1, agouti-related protein (AGRP), proopiomelanocortin (POMC), melanocyte-stimulating hormone (MSH), melanocortin, aggression, behaviour, fish

## Abstract

**Simple Summary:**

Enhanced feeding can be associated with aggressive behaviour since food resources are the main reason for agonistic behaviour (any social behaviour related to fighting). The overexpression of the gene agouti-signalling protein (Asip1) in transgenic zebrafish (*asip1-Tg*) results in enhanced food intake and linear growth. Our next question was if *asip1-Tg* animals exhibit a dominant phenotype associated with the feeding-enhanced levels when compared to wild-type (WT) fish. To address this question, we quantified the aggressive behaviour by conducting dyadic fights with real opponents as well as by exposing the animals to their specular image using mirrors. The results indicate that *asip1-Tg* are less aggressive than WT zebrafish in both dyadic fights and mirror-stimulus tests. These findings provide direct evidence of the role of the melanocortin system in the regulation of fish behaviour. The subordinate personality observed in *asip1-Tg* suggests that this transgene would be non-threatening to native populations in the event of an escape from aquaculture facilities. These results provide a genetic modification strategy to enhance growth in fish through high feeding motivation without promoting aggressiveness. This suggests that inhibiting the melanocortin system could be a viable target for genetically engineered fish. It is worth noting that the regulatory approval for such genetically engineered fish would be subject to the guidelines and regulations of the U.S. Food and Drug Association.

**Abstract:**

Feeding motivation plays a crucial role in food intake and growth. It closely depends on hunger and satiation, which are controlled by the melanocortin system. Overexpression of the inverse agonist agouti-signalling protein (ASIP) and agouti-related protein (AGRP) leads to enhanced food intake, linear growth, and weight. In zebrafish, overexpression of Agrp leads to the development of obesity, in contrast to the phenotype observed in transgenic zebrafish that overexpress *asip1* under the control of a constitutive promoter (*asip1-Tg*). Previous studies have demonstrated that *asip1-Tg* zebrafish exhibit larger sizes but do not become obese. These fish display increased feeding motivation, resulting in a higher feeding rate, yet a higher food ration is not essential in order to grow larger than wild-type (WT) fish. This is most likely attributed to their improved intestinal permeability to amino acids and enhanced locomotor activity. A relationship between high feeding motivation and aggression has been previously reported in some other transgenic species showing enhanced growth. This study aims to elucidate whether the hunger observed in *asip1-Tg* is linked to aggressive behaviour. Dominance and aggressiveness were quantified using dyadic fights and mirror-stimulus tests, in addition to the analysis of basal cortisol levels. The results indicate that *asip1-Tg* are less aggressive than WT zebrafish in both dyadic fights and mirror-stimulus tests.

## 1. Introduction

The melanocortin system is essential for regulating food intake, stress, and pigmentation in vertebrates [1]. Melanocortins are peptides derived from the post-transcriptional processing of proopiomelanocortin precursor (POMC), which encodes several melanocyte-stimulating hormones (MSHs) and the adrenocorticotropic hormone (ACTH) [2]. In tetrapod species, melanocortin signalling is mediated through five different receptors (MC1R-MC5R), which exhibit discrete functional domains and binding profiles [3]. MC2R is the only receptor exclusively activated by ACTH, whereas the remaining receptors bind the different MSHs with various affinities [3]. *Mc1r* is mainly expressed in the skin, where it binds α-MSH to regulate melanin synthesis and skin pigmentation. *Mc2r* is expressed in the adrenal cortex and mediates the stress response by triggering cortisol synthesis, which is released following ACTH binding. *Mc3r* and *Mc4r* are mainly expressed in the brain, where energy balance is regulated by binding MSHs. Inactivating mutations of MC4R result in enhanced linear growth and obesity in mice [4]. Finally, *Mc5r* is widely expressed at low levels and appears to regulate exocrine secretion in mice [5].

Atypically, the melanocortin system is also regulated by endogenous antagonists such as agouti-signalling protein (Asip) and agouti-related protein (Agrp). In mice, Asip regulates skin pigmentation by antagonising α-MSH effects on Mc1r in the follicle melanocytes. The *Ay* allele of the *asip* locus displays ubiquitous expression resulting in yellow fur and causing hyperphagia, obesity, and increased linear growth. Consequently, it emulates the metabolic phenotype of *mc4r (−/−)* mice [6]. The metabolic syndrome of *asip* overexpression is mediated by Mc4r, given that Asip can also antagonise MSH binding at Mc4r and depress the constitutive activity of Mc4r as an inverse agonist [7]. However, Asip is peripherally synthesised and only reaches the central nervous system (CNS) after ubiquitous expression. The central regulation of Mc4r signalling is modulated by Agrp, which is profusely expressed in the arcuate nucleus under fasting conditions. Accordingly, *agrp* transgenic mice exhibit a similar metabolic phenotype to that of the alleles *Ay* and *mc4r* (*−/−*) [8].

Teleost fish underwent an extra genome duplication event (TGD) that resulted in additional antagonist paralogues genes named *asip1*/*asip2* and *agrp1*/*agrp2*. Fasting increases agrp1 expression in the hypothalamus [9], and its overexpression in transgenic zebrafish (*Danio rerio*) promotes increased linear growth and weight [10]. Contrarily, *agrp1* morpholino knockdown fish show reduced growth mediated by MC4R constitutive activity [11]. Moreover, the genetic ablation of Agrp1 neurons results in decreased food intake in zebrafish [12]. Agrp2 appears to be involved in the stress response by suppressing interrenal cortisol release [12]. *asip1* is predominantly expressed in the ventral skin of goldfish (*Carassius auratus*) and zebrafish [13,14], thus regulating both melanogenesis and chromatophore fate [14,15] through Mc1r [16]. Recent studies have shown that *asip1* overexpression in transgenic zebrafish also results in increased food intake and linear growth, but no obesity phenotype [17]. Nevertheless, *asip1* transgenic fish do not require increased food intake to achieve a larger size. These transgenic fish, when fed at comparable rates to wild-type (WT) animals, demonstrate enhanced growth, indicating an enhanced food conversion rate [18]. A link between high feeding rates, enhanced growth, and dominance has been previously suggested in fish [19,20]. Growth hormone (*gh*) transgenic salmon exhibit increased food intake and growth rate and display pronounced dominant/aggressive behaviour [21,22]. Similarly, exogenous Gh administration promotes enhanced food motivation and increased growth in fish. However, it also results in increased aggression compared to WT animals [23,24]. In the present study, the potential of an *asip1-Tg* transgenic model is exploited in order to study the link between aggressiveness and enhanced feeding and growth rates, as well as the involvement of the melanocortin system in zebrafish behaviour.

## 2. Materials and Methods

### 2.1. Fish and Housing 

Wild-type (WT) and transgenic stocks come from the TU (Nüsslein-Volhard Lab, Tuebingen, Germany) strain. Generation of the transgenic zebrafish line [Tg(Xla.Eef1a1:Cau.Asip1]iim4, using the Tol2 transposon system, has been previously described [14]. Zebrafish embryos were obtained from natural crosses and kept at the Institute of Aquaculture of Torre de la Sal (IATS-CSIC) facilities at 27.5–28 °C under a 14 h/10 h light/dark cycle, with lights on at 7:00 a.m. and off at 9:00 p.m. They were raised in mixed-gender groups (∼50:50 male:female ratio) of ≈250 (fish) in 38 L tanks. Adult fish were fed a combination of artemia and commercial flake food (Vipan, Sera, Heinsberg, Germany) three times a day until satiety was reached. Commercial flake food has the following composition: 46.2% protein, 8.9% fat, 2.3% fibre, 11.9% ash, and 6.7% humidity. The animals used in this study were experimentally naïve and free of any signs of disease. They were tested only once, in a between-subject design, and returned to the stock tanks, remaining there for future experiments and breeding. 

### 2.2. Behavioural Experiments

Experiments were performed on 3- to 6-month-old male adult fish. Each test was performed on an independent cohort of 20 WT and 20 *asip1-Tg* fish (length WT = 25.2 ± 0.34; length *asip1-Tg* = 25.0 ± 0.19). Age and size were carefully paired among fish of different genotypes. At least one and a half weeks before assays, fish were transferred to a behavioural room in order to acclimatize. All behavioural assays were performed between 9:30 a.m. and 1:30 p.m. The different genotypes were intermixed throughout the experimental period to account for possible diurnal variations in behaviour. The fish were supplied with food 30 min prior to the beginning of the trials.

Two commonly used methods were employed to quantify aggressive behaviour in zebrafish. Such methods are based on two paradigms: (1) the dyadic fight test between real opponents, WT and *asip1-Tg*; and (2) mirror stimulus tests. The protocols applied were essentially those published by [25]. The testing apparatus consisted of a 26 cm × 15 cm × 17 cm tank divided into two compartments, with one division containing a mechanical filter and a heater (set to conserve water temperature at 28 °C). The test was conducted in the other compartment, which was equipped with a perforated back wall covered with white-coloured self-adhesive film to improve contrast. Additionally, it was outfitted with a removal partition, creating two identical experimental tanks. Each tank was equipped with a mirror that was covered by a sliding, opaque shield. Subjects were consistently tested in pairs in order to provide them with conspecific odours, which would otherwise only be present in real-opponent dyads (see Figure S1 in Supplementary Materials).

The identification of individuals involved in dyadic fights was carried out based on pigmentation differences among genotypes [14], so tagging was unnecessary. Animals of each genotype were paired according to their standard length. Differences in length between opponents did not exceed 2% of the total body size. The fish remained visually isolated overnight in the experimental tank. The following day, the partitions were removed, allowing the fish to interact for 30 min, a duration that exceeded the time required to determine a clear winner of the contest. Following each interaction, the fish were once again separated by replacing the opaque partition. For the mirror–image stimulus, naïve fish (n = 20) were allowed to acclimate overnight as previously described, and the sliding opaque shields were removed. This allows the fish to interact with their specular image for 25 min.

### 2.3. Quantification of Aggressive Behaviour

Recording of the fish activity was performed via industrial digital cameras from IDS (UI-3240CP USB 3.0 uEye CP, IDS Imaging Development Systems GmbH, Obersulm, Germany) and/or Basler (Basler acA1280-60gc GigE camera, Basler AG, Ahrensburg, Germany) equipped with a high-quality monofocal lens (focal length 8 mm) with a frame acquisition rate of 25 fps. Behavioural event logging was carried out using the free open-source software BORIS [26], while animal trajectory tracking was performed using EthoVision^®^ XT software (Noldus Inc., Wageningen, The Netherlands). With regard to staged fights, the latency and direction (who attacked whom) of the first territory incursion by the opponent were assessed. Behavioural interaction was analysed by an experienced observer in order to identify relevant agonistic behaviours classified as aggressive for dominant fish (bite, chase, strike) and submissive for subordinates (flee and freeze), according to the published ethogram of aggressive behaviour of male zebrafish [27]. Based on who attacks and who is submissive in the post-resolution phase of the interaction, the winner and loser of each fight were determined. For the analysis of the mirror–image stimulus test, the arena of each individual was delimited into three zones: (a) a safe area consisting of the bottom part of the tank at a distance from the mirror; (b) a near area to the mirror, excluding the mirror itself; and (c) a close area where direct contact of the fish with the mirror could take place, either by mouth or the lateral part of the body. To determine differences in risk assessment between genotypes, the latency to both the first exploration and interaction and the latency to repeat such behaviour were measured. The percentage of time spent by each genotype in each of the three areas was also measured in order to quantify the amount of time spent interacting with their specular image. Other measurements included the number of interactions and explorations per minute at the exploration and interaction areas, respectively, and the meantime employed per interaction. Furthermore, the frequency of typical overt aggressive behaviours, such as attempted bites at the mirror reflection, lateral display, charge, and frontal and parallel swimming while maintaining contact with the mirror using the snout, as well as the duration of such behaviours, were manually assessed for 8 pairs of fish for 10 min [25,28,29]. Ultimately, the mean distance to the mirror and the number of 360° rotations per area were measured.

### 2.4. Cortisol Determination

Ten fish of each genotype (50:50 male–female) were placed in two tanks (10 L) for 30 days and sampled for whole-body cortisol determination [30]. The fish were euthanised by immersion in ice-cold water containing a buffered solution of tricaine methanesulfonate (MS-222, Sigma-Aldrich, Burlington, MA, USA) at a concentration of 200 μg/mL. Euthanasia was considered complete when the fish turned upside down, their eyes and operculum become immobile, and no response was observed in their tails upon contact. Subsequently, the fish were stored intact at −80 °C. Prior to hormonal determination, the fish were thawed and weighed, and 0.5 mL of ice-cold 1X PBS (phosphate-buffered saline) was added prior to homogenisation using a Polytron^®^ PT 1200 E (Kinematica, Malters, Switzerland). Cortisol extraction was carried out by adding 5 mL of diethyl ether (Fisher Scientific International, Inc., Waltham, MA, USA). After 20 min, the samples were centrifuged at 300× *g* and the organic upper layer was transferred to new glass tubes. The extraction protocol was repeated by adding 2 mL of fresh diethyl ether to ensure optimal cortisol recovery. Subsequently, evaporation was carried out on the samples using a speed vacuum centrifuge. Cortisol levels were quantified by enzyme immunoassay (EIA, Cayman Chemical Company, Ann Arbor, MI, USA) using a 200Pro plate reader (TECAN, Grödig, Austria) and standardised according to the weight of the fish (ng cortisol/g fish). 

### 2.5. Statistical Analysis

Statistical analyses were performed using the GraphPad Prism 8 software (GraphPad Software Inc., San Diego, CA, USA). For the dyadic fights, an unpaired t-test was used to compare the latency for the first territory incursion between genotypes. Fisher’s exact test was used to compare differences in the first bite performed by individual fish between genotypes. The number of dominant, subordinate, or undetermined fish was reported per genotype, and results were analysed by the Chi-square test.

In the mirror–image stimulus test, differences in the latency to both the first visit and the latency to revisit the close and near zones to mirror those behaviours, 360° rotations per zone were analysed by a repeated measures mixed-effects model followed by Sidak’s multiple comparison test. The time each fish spent in each area and the visit velocities, expressed as the number of entries per minute spent close to or near the mirror, were studied using a two-way ANOVA with repeated measures, followed by Sidak’s multiple comparison test. An unpaired t-test was employed to investigate differences in the meantime per interaction, the mean distance to the mirror, and the number of lateral displays, charges, frontal swimming, and bites. This method was also applied to compare the frequency of charges, bites, lateral displays, and the duration of frontal swimming. An unpaired t-test was also used to compare the cortisol levels between *asip1-Tg* and WT fish. All data are presented as mean ± SEM, and the significance level was set at *p* < 0.05. 

## 3. Results

### 3.1. Agonistic Behaviour in Zebrafish

Aggressiveness serves various adaptive functions, such as the establishment of dominance relationships and hierarchies and competition for key resources such as food, shelter, mates, or territories. Zebrafish are a gregarious species that exhibit shoaling behaviour in captivity. However, several studies have previously demonstrated that both male and female fish exhibit aggressive behaviour [27,31]. In the present study, dyadic fights between size-paired males were used in order to examine aggressiveness in transgenic zebrafish, which overexpress an antagonist of the melanocortin system. Male zebrafish dyads follow stereotyped behavioural patterns, with a well-structured temporal pattern that has been previously and thoroughly characterised, thus allowing for standardisation [27]. 

**Figure 1:**
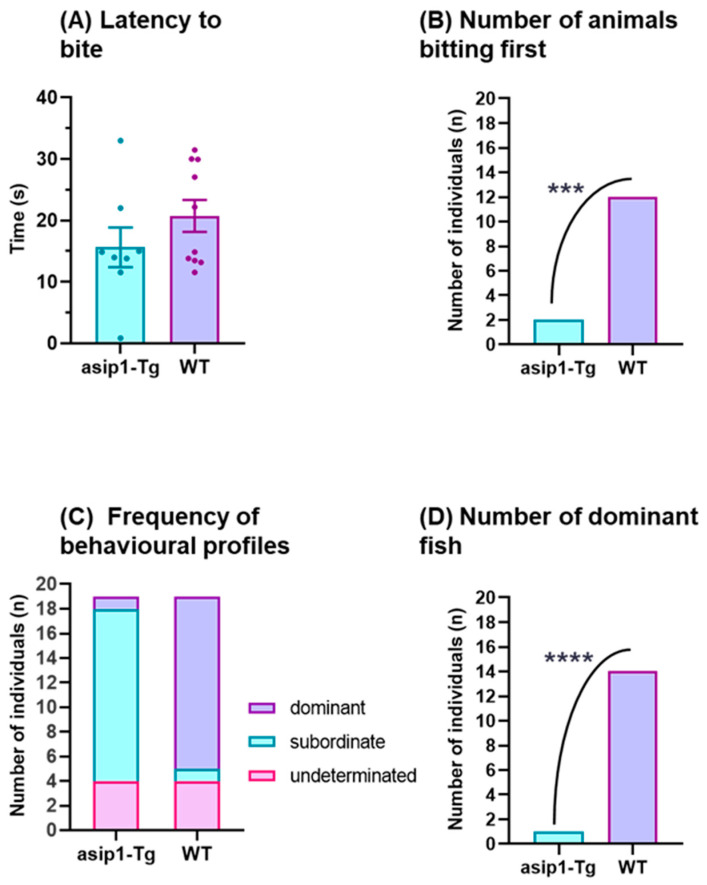
Behavioural differences between transgenic (*asip1-Tg*) and wild-type (WT) zebrafish in dyadic fights. (**A**) Latency to the first bite, (**B**) Number of animals biting first, (**C**) frequency of behavioural profiles, and (**D**) number of dominant fish. Experiments were performed on 20 WT and 20 *asip1-Tg* fish paired by age and size. Data are represented as mean ± SEM and analysed by unpaired *t*-test (latency to first territory intrusion) or as the number of fish and analysed by Chi-square test (dominant fish) or Fisher’s exact test (first to bite). Asterisks indicate statistical differences between genotypes (*** *p* ≤ 0.001, **** *p* ≤ 0.0001).

During the experimental period of this study, two phases were easily differentiated. The initial phase consisted of mutual assessment behaviours, with fish circling and biting each other in order to determine the relative fighting ability of the opponent. Such a phase started with the first interaction of the trial and ended when the first chase/retreat was observed, thus marking the resolution point of the fight. Since the bite is the most frequent behaviour, representing over 50% of all behaviour types exhibited by zebrafish in the first phase. It was decided to measure which genotype bites, it was found that WT significantly bit first (*p* = 0.0019) (Figure 1A), although there were no significant differences in latency to the first territory intrusion (*p* = 0.2291) (Figure 1B). During the second phase, which is characterised by chasing and retreating until the subordinate fish typically falls into a freeze-type behaviour, *asip1-Tg* fish fled when chased by WT animals, consequently remaining immobile in one corner at the bottom of the tank (freezing behaviour). Approximately 74% (14 out of 20) of *asip1-Tg* fish exhibited the aforementioned behaviour during dyadic fights. Transgenic fish only exhibited dominant behaviour in one of the experimental conflicts, and four dyadic pairs showed no apparent agonistic behaviour (Figure 1C). The results show an evident subordinate personality in *asip1-Tg* (*p* < 0.0001) (Figure 1D). 

**Figure 2:**
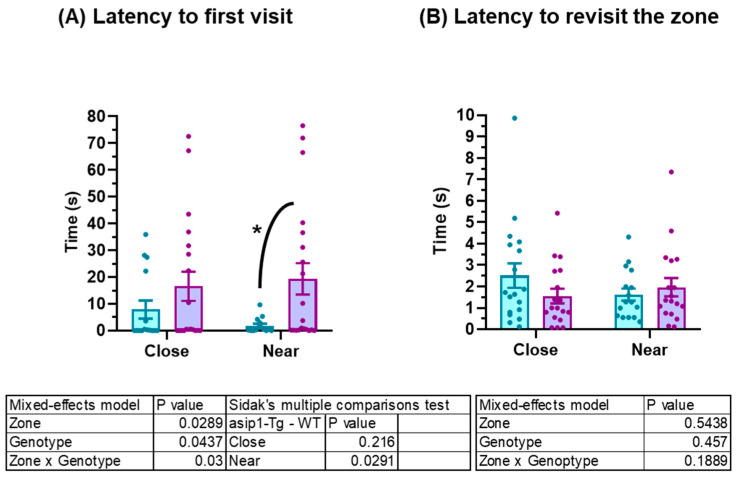
Behavioural differences between *asip1-Tg* and WT in the mirror–image stimulus test. (**A**) Latency to both first approximation (close area) and exploration (near area); (**B**) Latency to repeat approximation and exploration behaviours. Experiments were performed on 20 WT and 20 *asip1-Tg* fish. Data are represented as mean ± SEM and analysed by two-way ANOVA repeated measures (RM), followed by Sidak’s multiple comparison test. Blue and purple colours represent *asip1-Tg* and WT fish, respectively. Asterisks indicate statistical differences between genotypes (*p* ≤ 0.05).

Agonistic behaviour was also quantified by using the mirror–image stimulus. Fish fail the self-recognition test, thus attacking the image reflected in the mirror as a conspecific or rival [32,33]. Significant differences were found in the latency to the first visit; specifically, *asip1-Tg* entered the zone near the mirror first than WT, but they did not differ in obtaining the zone close to the mirror area in terms of time (Figure 2A). Nevertheless, no differences between genotypes were found in the latency to revisit either of the zones (Figure 2B). 

**Figure 3:**
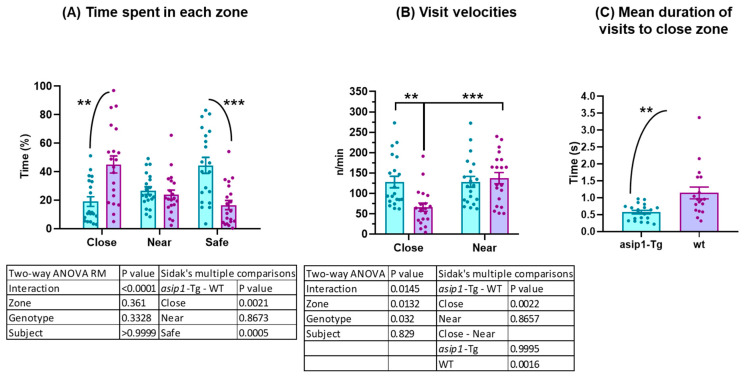
Behavioural differences between *asip1-Tg* and WT in the mirror–image stimulus test. (**A**) Time spent in three previously defined areas: (i) a safe area consisting of the bottom part of the tank; (ii) an area near the mirror; and (iii) an area close to the mirror where direct contact of the fish with the mirror may take place. (**B**) Visits’ velocity is computed as the number of entries per minute spent near or close to the mirror. (**C**) Mean duration of visits to the close area. Experiments were performed on 20 WT and 20 *asip1-Tg* fish. Data are represented as mean ± SEM and analysed by two-way ANOVA repeated measures (RM) followed by Sidak’s multiple comparison tests for (**A**,**B**) or unpaired *t*-test for (**C**). Asterisks indicate statistical differences between genotypes (** *p* ≤ 0.01, *** *p* ≤ 0.001). Blue and purple colours represent *asip1-Tg* and WT fish, respectively.

Transgenic fish spent significantly less time close to the mirror and more time in the safe area (Figure 3A). Surprisingly, the visits’ velocity, e.g., entries per minute spent in an area, was significantly higher close to the mirror in *asip1-Tg* than in WT fish (Figure 3B). Despite this, WT animals spent double the time close to the mirror in each visit compared to transgenic fish (*asip1-Tg* mean = 0.571 s, WT mean = 1.143 s; *p* = 0.0022) (Figure 3C). Significant differences in visits’ velocity between zones close to and near the mirror were only found in the WT group (*p* = 0.0016) (Figure 3B). Taken together, these results suggest a significant difference in fighting behaviour between genotypes. While *asip1-Tg* preferred the safe area, WT fish confronted their specular image for longer periods of time close to the mirror, contrary to transgenic animals, which approach the mirror with a higher frequency but for a shorter period of time.

**Figure 4:**
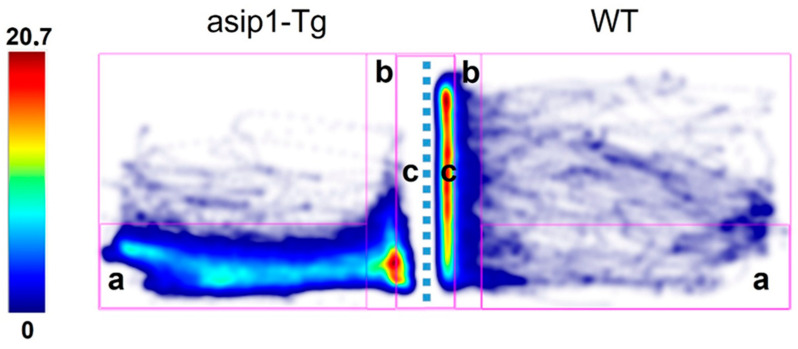
Representative heatmaps of *asip1-Tg* (**left**) and WT (**right**). The colour scale represents the cumulative time spent in each of the previously defined arena zones. (a) a safe area consisting of the bottom part of the tank; (b) near the mirror zone, an exploration area where fish can approach but not touch the mirror; and (c) a close to the mirror area where direct contact of the fish with the mirror can take place. The dotted line represents the position of the mirror.

Such behaviour is reflected in the heatmap analysis, where the colour patterns show that *asip1-Tg* fish appear to flee from their reflection in the mirror (Figure 4). It is also supported by the higher number of rotations per minute displayed by *asip1-Tg* in the safe area and the almost double mean distance from the mirror kept by *asip1-Tg* compared to WT (see also Figures S2 and S3 in Supplementary Materials).

**Figure 5:**
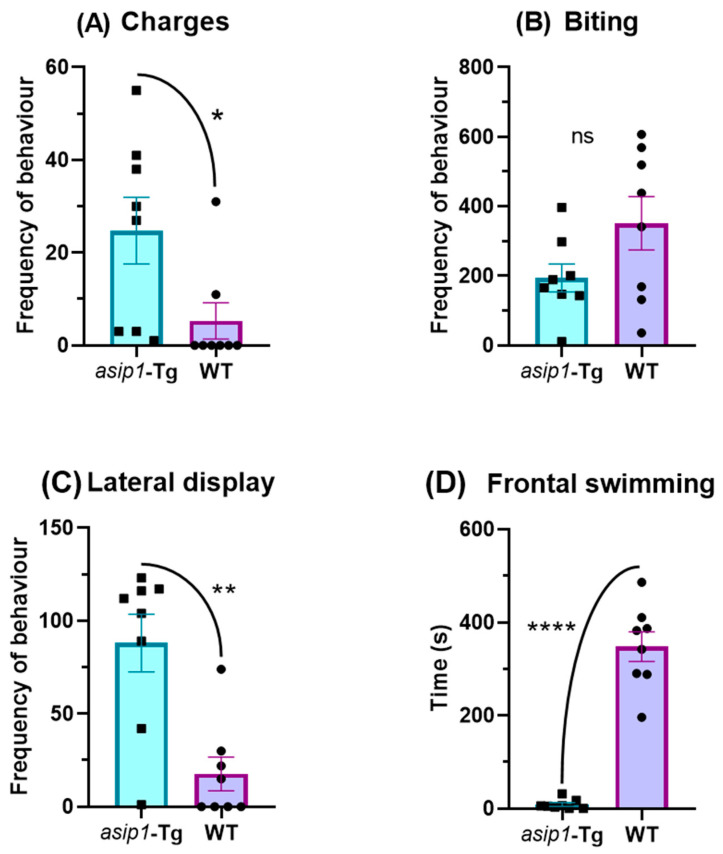
Frequency and duration of aggressive-related behaviours of *asip1-Tg* and WT in the mirror-stimulus test. (**A**) Charges; (**B**) biting; (**C**) lateral display; (**D**) frontal swimming. Data relate to 8 WT and 8 *asip1-Tg* fish and are represented as mean ± SEM. Data were analysed by an unpaired t-test. Asterisks indicate statistical differences between genotypes (* *p* ≤ 0.05, ** *p* ≤ 0.01, **** *p* ≤ 0.0001). ns indicates non-significant differences.

The frequency of WT overt aggressive behaviour was higher than that observed in *asip1-Tg*, with WT fish attempting to bite their reflection more frequently than *asip1-Tg*, although not statistically different (*p* = 0.0908) (Figure 5B). WT also performs frontal and parallel swimming, maintaining contact with the snout to the mirror significantly longer than *asip1-Tg* (*p* < 0.0001) (Figure 5C,D). On the contrary, the frequency of restrained aggressive behaviour events, charges, and lateral display, behaviours with no physical contact, was significantly higher in *asip1-Tg* fish than in WT (*p* = 0.0321 and *p* = 0.0015, respectively) (Figure 5). Such findings provide evidence, which once again, reinforces the subordinate personality of *asip1-Tg* zebrafish.

### 3.2. Whole-Body Cortisol Levels

**Figure 6:**
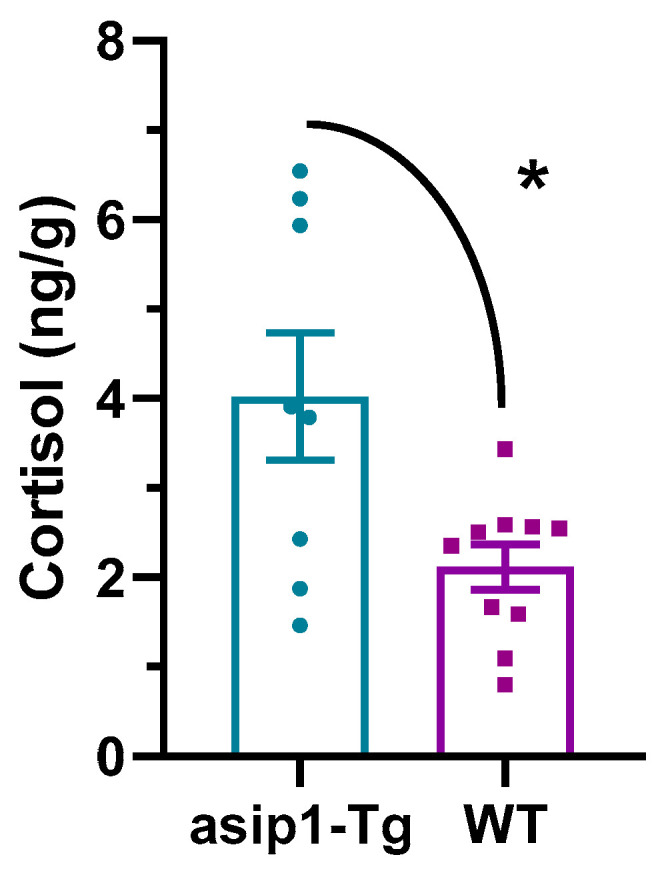
Whole-body basal cortisol levels in *asip1-Tg* and WT zebrafish. Ten fish of each genotype were homogenised, and cortisol was extracted and quantified by EIA. An asterisk indicates statistical differences between genotypes (* *p* terisk i).

Whole-body cortisol basal levels in both genotypes were determined by EIA assays. It was observed that *asip1-Tg* zebrafish exhibited significantly higher total cortisol levels than WT animals (*p* = 0.0146) (Figure 6). 

## 4. Discussion

Previous studies have demonstrated that *asip1* overexpression in a transgenic model promotes a profuse change in the dorsoventral pigment pattern in zebrafish [14,15]. In addition, *asip1-Tg* fish exhibit higher food intake levels, which result in a greater growth rate, yet animals are found not to be obese. Such increased food intake appears to be the result of a desensitised satiety system that promotes enhanced feeding motivation [17,18]. Although *asip1-Tg* zebrafish grow faster, puberty is not reached in advance, as the effects of the transgene on growth are only noticeable after a threshold length when puberty has already been attained [34]. It is therefore assumed that, being larger, *asip1-Tg* fish could compete more efficiently for food while exhibiting aggressive behaviour. Indeed, aggression serves several adaptive functions, including the competition for key resources such as food, territories, or mates [27]. Live conspecific and mirror–image stimuli were used to enable the characterization of agonistic behaviour in transgenic animals. As zebrafish are territorial, this simple behavioural paradigm, in which fish are pre-exposed to 16 h of social isolation, promotes fighting behaviour, even in the presence of abundant, not-limiting resources [27]. The results demonstrate that while both genotypes of fish tend to invade the territory of others without significant differences in risk assessment, the *asip1-Tg* fish display subordinate behaviour in dyadic interactions with WT animals. Out of the 20, transgenic fish tested, only one emerged as the dominant winner, highlighting their overall subordinate status in these interactions. The outcome of the behaviour observed during the conspecific fights was similar to the aggressive behaviour expressed towards a mirror in the mirror–image stimulus test. Subjects of the *asip1-Tg* genotype spent significantly less time interacting with their specular images and more time than WT in the safe area, thus avoiding their reflection in the mirror. Such observations reflect cautious and elusive behaviour under a given potential risk. Even though *asip1-Tg* reacted against their specular image, they carried out significantly more restrained aggressive displays, charges, and lateral displays, behaviours that display no physical attacks. On the other hand, WT fish maintained contact with their snout toward the mirror for most of the experimental period and attempted to bite their reflection more frequently than *asip1-Tg*, although no significant differences were observed. On the whole, results from the mirror–image stimulus corroborate the submissive phenotype of *asip1-Tg* fish. While the mirror–image test is a standardised assay for quantification of aggressive behaviour, the physiological underpinnings are not completely reproduced, particularly at the level of the central transcriptome and/or endocrine system and behavioural responses [28,35,36]. In fact, a true subordinate phenotype, as observed in real-opponent fights, is never exhibited in this case. The outcome of the fight does not occur, and the expression of aggressiveness is uncoupled from the experience of the contest result [35]. Nevertheless, the total level of aggressiveness and the stereotyped components of the aggression between the males exposed to real and mirror–image opponents were similar in *Astatotilapia burtoni* [28] and zebrafish [37]. A recent report showed that intraperitoneal administration of α-MSH increased the rate of aggressiveness, while mammalian Asip reduced it in the cichlid fish species *Astatotilapia burtoni* [38]. These findings align with our results and further support the role of the melanocortin system in the modulation of agonistic behaviour in fish. 

Although *asip1-Tg* zebrafish grow faster and longer than WT siblings [17,18], the differences observed cannot be attributed to the size of the animals, as opponents were paired by body length. The ages of both genotypes were also paired. Therefore, the reproductive status was similar for both genotypes. The diminished amount of time of interaction with the mirror could also arise from the reduced locomotor activity of the transgenic animal stemming from genetic manipulation. However, no differences were observed in the total distance travelled or mean velocity between phenotypes (data not shown). Recent studies have suggested that subordinate animals are capable of adapting their behavioural output to novel social contests in order to reduce energetic expenditure and lower the risk of injuries and exhaustion. They achieve this by exhibiting submissive behaviour, such as behavioural inhibition, particularly under increased dominance threats [39,40]. In the present experiment, encounters between *asip1-Tg* and a WT conspecific showed predominantly unidirectional aggression from WT fish towards *asip1-Tg*. Twelve WT fish out of twenty bit the opponent first, while only two *asip1-Tg* attacked first. Thus, *asip1-Tg* fish were found not to challenge the WT fish for dominance and instead decided to flee or remain motionless in the bottom corner. It can be argued that the social competence of *asip1-Tg*, defined as an individual’s ability to use social information in order to optimise its social behaviour [39,40], allows such fish to conserve energy (by avoiding the fight for dominance) and evade aggression. In such a context, it is noteworthy that being subordinate may be costly for the individual, as it is associated with low activity levels, reduced growth, suppressed feeding, and reduced reproduction [41,42,43]. Nevertheless, such negative outcomes are unobserved in the *asip1-Tg*, as overexpression of Asip1 results in increased food intake and linear growth. Furthermore, our unpublished results indicate that *asip1-Tg* fish exhibit increased locomotor activity during the circadian period, particularly during the night periods (Godino-Gimeno A, Puchol S, Rocha A, and Cerdá-Reverter JM). In all likelihood, this is a result of increased foraging behaviour due to the desensitised satiety system [17]. 

*asip1* transgene overexpression yields a paler colour of dorsal pigmentation in zebrafish [14]. It cannot entirely be disregarded that our behavioural results are, to some extent, due to differences in the dorsoventral pigmentation pattern. In fact, fish coloration is an important visual signal linked to aggressive behaviour and/or social rank used by territorial animals [44]. Therefore, the WT animals interpret the paler dorsal pigmentation of *asip1-Tg* as a marginal phenotype associated with a suboptimal physiological condition. This perception provides a chance for the opponent to reach social dominance. Under this hypothesis, *asip1-Tg* animals should exhibit similar aggressive levels to those of the WT animals in the mirror-stimulus test. In this test, the image stimulus provided corresponds to a paler animal, and since fish are unable to self-recognise [36] (see also [45,46,47] for controversial discussion), *asip1-Tg* animals would perceive the reflected image as a paler individual as well. However, despite the similarity in the reflected image, transgenic fish display a submissive-like behaviour in front of the mirror, which is suggested by the reduced interaction with their own image. It can therefore be argued that the dorsoventral pigment pattern did not affect behaviour outcome.

Endocrine and neuroendocrine systems play a crucial role in the regulation of social behaviour, including aggressiveness and the acquisition of social status (dominance vs. submission) in fish [44,48,49,50]. However, the involvement of the melanocortin system in the regulation of social behaviour has been scarcely studied. The neuronal mechanisms responsible for the behavioural phenotype behind *asip1* overexpression are unknown. It is widely recognised that Mc4r agonists improve the social deficits observed in NFK1−/− mice through the oxytocin pathway [51]. However, it is worth noting that central administration of α−Msh has been shown to decrease social rewards in an oxytocin-dependent manner in Syrian hamsters (*Mesocricetus auratus*) [52]. Previous experiments in goldfish [53], sea bass [54], and zebrafish [55] have demonstrated that MC4R is profusely expressed in the parvocellular and magnocellular preoptic areas as well as within the whole extension of the tuberal hypothalamus. The diencephalic region of fish produces two nonapetides, namely isotocin (It, an analogue to mammalian oxytocin) and arginine vasotocin (Avt, an analogue to the mammalian arginine vasopressin). These nonapeptides play critical roles in the social behaviour of fish, including aggressiveness [48,49,56]. Dominant zebrafish exhibit more Avt magno- and giganto-cellular neurons of the preoptic area (POA) after dyadic tests, while submissive or loser animals display an abundant presence of Avt neurons in the parvocellular POA [57]. In other species, such as *Oreochromis mossambicus*, the aggressive behaviour correlates to the number of Avt cells in the parvocellular region, whereas the social submission is associated with changes in the Avt cell populations in the magno- and giganto-cellular preoptic neurons [58,59]. Therefore, teleost brains have multiple nonapeptidergic pathways that modulate social responses associated with parvo and magnocellular neurons of the POA. 

It can be assumed that melanocortins modulate Avp availability in the preoptic region via Mc4r, thus promoting a submissive phenotype. The regulation of the diencephalic nonapeptidergic system by the melanocortin system has already been reported in rats. Central administration of the MC3/4R agonist melanotan II (MTII) increases c-Fos expression in AVP neurons of the paraventricular hypothalamus (PVH), a mammalian homologue of the non-tetrapod magnocellular preoptic nucleus [60], to acutely inhibit food intake. In addition, MTII fails to fully suppress feeding in mice with virally-mediated PVH-AVP neuron ablation [61]. Therefore, it can be assumed that preoptic Mc4r could induce Avt expression in the parvocellular nucleus and/or reduce the number of Avt neurons in the magnocellular region to promote submissive behaviour and modulate food intake levels. Melanocortin-induced modulation of social behaviour would be modulated by the parvocellular Avt neurons, whereas food intake levels would be modulated by magnocellular Avt neurons. In fact, intracerebroventricular injections of Avt inhibit food intake and induce anxiety-like behaviour in goldfish and rainbow trout [62,63]. Suggestively, our unpublished results demonstrate that *asip1-Tg* animals display anxiety-like behaviour after several behavioural assays, including the novel tank test. However, the effects of Asip1 appear to be mediated via the serotoninergic system.

The submissive behaviour of *asip1-Tg* animals could be part of a behavioural adjustment in order to reduce their stress load, as transgenic zebrafish also showed increased whole-body cortisol levels compared to WT fish. Subordinate individuals in groups with stable hierarchies, in which dominance is attained by aggression and intimidation, show the greatest physiological indices of stress [64]. Therefore, such individuals need to adjust their behaviour, such as adopting submissive behaviour, in order to reduce their stress load. 

Proactive and reactive animals respond differently to stressful events, with proactive animals being more aggressive and mobile, yet also having a lower hypothalamic-pituitary-interrenal axis (HPI, the fish homologue of the tetrapod hypothalamic-pituitary-adrenal axis, HPA). On the contrary, reactive/submissive animals exhibit higher HPI activity, which leads to increased cortisol levels. The associated genotypes can be genetically segregated into different lines as high/proactive (HR) and low/reactive (LR) strains [65,66]. Numerous studies have shown that individuals who respond to stress with high HPA/HPI axis reactivity (the mammalian equivalent to the HPI axis in fish) are less aggressive than those who respond with lower HPA axis reactivity. Accordingly, HR rainbow trout displayed more aggressiveness than LR strains in resident–intruder tests [67]. Our previous experiments in zebrafish have shown that whole-body cortisol levels can be a good indicator of HPI axis activity [32], providing more support for the submissive behavior of the *asip1-Tg* genotype.

## 5. Conclusions

In conclusion, results show that the reduced melanocortin signalling imposed by the overexpression of endogenous antagonists leads to less aggressiveness in animals, thus suggesting a submissive phenotype. Behavioural phenotype is in accordance with physiological data, as *asip1-Tg* fish also display higher cortisol levels, which is distinctive of reactive/submissive phenotypes. These results imply direct evidence of the role of the melanocortin system in the regulation of fish behaviour and provide new central mechanisms for future behavioural studies.

## Data Availability

Data are available at https://data.mendeley.com/datasets/999v2k53fv/draft?a=ba0ce768-20be-4782-acc3-ba32b2dfe2db (accessed on 14 February 2023)).

## Supplementary Materials

The following supporting information can be downloaded at: https://www.mdpi.com/article/10.3390/biology12050712/s1, Figure S1: Mean distance to mirror of asip1-Tg (n = 20) and WT (n = 20) in the mirror-image stimulus test. Figure S2: 360° Rotations per minute in the mirror-image stimulus test.
