# Supplementary material for: Agouti-Signalling Protein Overexpression Reduces Aggressiveness in Zebrafish
Source: Biology (Basel). 2023 May 13;12(5):712. doi: 10.3390/biology12050712 (PMC10215356; doi:10.3390/biology12050712)
Supplement: Supplementary file 1 [file biology-12-00712-s001.zip › biology-2273978-supplementary.pdf]

biology

MDPI

SUPPLEMENTARY INFORMATION

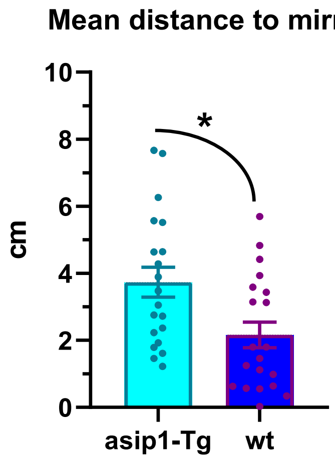

Figure S1. Mean distance to mirror of *asip1*-Tg (n = 20) and WT (n = 20) in the mirror-image stimulus test. Data were represented as mean ± SEM and analysed by Unpaired t-test. Asterisks indicate statistical differences between genotypes (*p ≤ 0.05). Blue and purple colours represent *asip1*-Tg and WT fish, respectively.

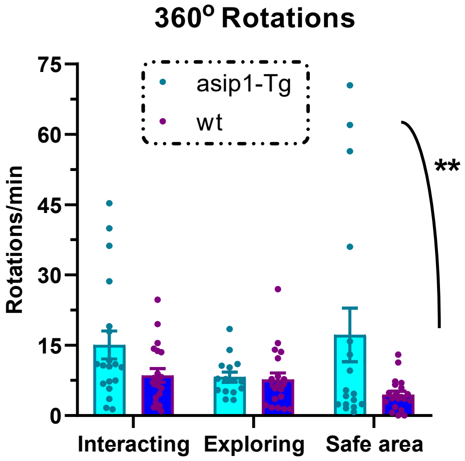

Two-way ANOVA	P value	Sidak's multiple comparisons test	
Interaction	0,0868	asip1-Tg - wt	P value
Behaviour	0,3456	Interacting	0,2224
Genotype	0,0035	Exploring	0,9991
		Safe area	0,0034

Figure S2. 360º Rotations per minute in the mirror-image stimulus test. Three arena zones were previously defined i) a safe area, consisting of the bottom part of the tank, ii) an exploration zone close to the mirror and iii) an interaction zone where direct contact of the fish with the mirror can occur. Experiments were performed on 20 WT and 20 *asip1*-Tg fish. Data are represented as mean ± SEM and analysed by two-way ANOVA followed by Sidak's multiple comparison test (latency to first). Asterisks indicate statistical differences between genotypes (*p ≤ 0.05, **p ≤ 0.01).
